# Effects of irrigation and nitrogen application on soil water and nitrogen distribution and water-nitrogen utilization of wolfberry in the Yellow River Irrigation Region of Gansu Province, China

**DOI:** 10.3389/fpls.2023.1309219

**Published:** 2023-12-11

**Authors:** Rongrong Tian, Guangping Qi, Yanxia Kang, Qiong Jia, Jinghai Wang, Feng Xiao, Yalin Gao, Chen Wang, Qiang Lu, Qidong Chen

**Affiliations:** ^1^ College of Water Conservancy and Hydropower Engineering, Gansu Agricultural University, Lanzhou, China; ^2^ Jingtaichuan Electric Power Irrigation Water Resource Utilization Center in Gansu Province, Baiyin, China

**Keywords:** water and nitrogen regulation, soil moisture content, soil NO_3_^−^–N, yield, water-nitrogen use efficiency, wolfberry

## Abstract

To address the problems of extensive field management, low productivity, and inefficient water and fertilizer utilization in wolfberry (*Lycium barbarum* L.) production, an appropriate water and nitrogen regulation model was explored to promote the healthy and sustainable development of the wolfberry industry. Based on a field experiment conducted from 2021 to 2022, this study compared and analyzed the effects of four irrigation levels [75%–85% θ_f_ (W0, full irrigation), 65%–75% θ_f_ (W1, slight water deficit), 55%–65% θ_f_ (W2, moderate water deficit), and 45%–55% θ_f_ (W3, severe water deficit)] and four nitrogen application levels [0 kg·ha^−1^ (N0, no nitrogen application), 150 kg·ha^−1^ (N1, low nitrogen application), 300 kg·ha^−1^ (N2, medium nitrogen application), and 450 kg·ha^−1^ (N3, high nitrogen application)] on soil water distribution, soil nitrate nitrogen (NO_3_^−^–N) migration, yield, and water-nitrogen use efficiency of wolfberry. The soil moisture content of the 40–80 cm soil layer was higher than those of 0-40 cm and 80-120 cm soil layer. The average soil moisture content followed the order of W0 > W1 > W2 > W3 and N3 > N2 > N1 > N0. The NO_3_^−^–N content in the 0–80 cm soil layer was more sensitive to water and nitrogen regulation, and the cumulative amount of NO_3_^−^–N in the soil followed the order of W0 > W1> W2 > W3 and N3 > N2 > N1 > N0 during the vegetative growth period. There was no evidently change in soil NO_3_^−^–N accumulation between different treatments during the autumn fruit. The yield of wolfberry under the W1N2 treatment was the highest (2623.09 kg·ha^−1^), which was 18.04% higher than that under the W0N3 treatment. The average water consumption during each growth period of wolfberry was the highest during the full flowering period, followed by the vegetative growth and full fruit periods, and the lowest during the autumn fruit period. The water use efficiency reached a peak value of 6.83 kg·ha^−1^·mm^−1^ under the W1N2 treatment. The nitrogen uptake of fruit and nitrogen fertilizer recovery efficiency of fruit first increased and then decreased with increasing irrigation and nitrogen application. The treatment of W1N2 obtained the highest nitrogen uptake of fruit and nitrogen recovery efficiency of fruit, which were 63.56 kg·ha^−1^ and 8.17%, respectively. Regression analysis showed that the yield and water-nitrogen use efficiency of wolfberry improved when the irrigation amount ranged from 315.4 to 374.3 mm, combined with nitrogen application amounts of 300.0 to 308.3 kg·ha^−1^. Additionally, the soil NO_3_^−^–N residue was reduced, making it an optimal water and nitrogen management model for wolfberry planting. The present findings contribute novel insights into the production of wolfberry with saving water and reducing nitrogen, which helps to improve the level of wolfberry productivity in the Yellow River irrigation region of Gansu Province and other areas with similar climate.

## 1 Introduction

Wolfberry (*Lycium barbarum* L.) is a perennial deciduous shrub belonging to the Solanaceae family (Kulczyński and Gramza-Michałowska, 2016). It thrives in cold and cool climates and exhibits strong resistance to cold temperatures (Yao et al., 2018). It plays an important role in medical health care, water and soil conservation, and saline-alkali soil improvement (Wozniak et al., 2012; Danial et al., 2022). Wolfberry is mainly distributed in China, the United States, France, and other places, among which China is the main producer of wolfberry in the world, and the planting area accounts for approximately 95% of the total global planting area (Anastasia et al., 2014; Huang and Zhang, 2021). Since the “14th Five-Year Plan”, under the background of structural reform on the agricultural supply side and characteristic agricultural products driving local economic development, wolfberry has become an advantageous industry in northwest China. By 2020, the planting area of wolfberry will reach 148,300 ha, and the output of dried fruit will be approximately 428,500 tons (Ma et al., 2021; Hao, 2022). The rapid development of the wolfberry industry is of great significance for alleviating the problems of the “three rural areas”, consolidating the achievements of poverty alleviation, and promoting rural revitalization (Jing and Han, 2018; Ren and Wang, 2019; Wang et al., 2021). However, the northwest of China is short of water resources and poor in soil (Zhang et al., 2015). The extensive management of water and fertilizer in the cultivation of wolfberry not only reduces crop productivity and utilization of water and fertilizer resources, but also increases greenhouse gas emissions in farmland, and aggravates greenhouse effect (Lu et al., 2022). At the same time, the excessive irrigation and fertilization will also leading to soil aeration and water permeability, affecting crop nutrient absorption and normal growth (Zhou and Bai, 2015), raising the groundwater level, increasing the salt content of soil solution and the decomposition of soil organic matter, causing groundwater pollution, soil salinization and soil acidification (Li et al., 2015a; Niu et al., 2022), and accompanied by problems of soil desertification and desertification (Chen and Tian, 2011). Therefore, exploring the development potential of appropriate water and fertilizer management modes in the wolfberry industry is very important. It will not only contribute to alleviate the problems of water and fertilizer resource waste and ecological environment pollution, but also contribute to improve the productivity of wolfberry and ensure the green and sustainable development of the wolfberry industry in northwest China and even the global.

Water and nitrogen are important factors that limit crop growth and yield (Gonzalez-Dugo et al., 2010; Darren et al., 2020; Tan et al., 2021). As a good solvent and carrier, water not only accelerates the dissolution and mineralization of nitrogen in soil but also transports it to crop roots in the form of diffusion or mass flow (Crevoisier et al., 2010), which affects the availability of soil nitrogen and changes the absorption, transport, and assimilation of nitrogen by crops (Guan et al., 2015). Nitrogen is a macronutrient in crops that significantly affects the accumulation and distribution of dry matter (Sun et al., 2014). The supply of water and nitrogen, either too high or too low, is detrimental to crop growth. The lack of soil water and nitrogen limits the potential for crop production. However, excessive soil water and nitrogen reduce soil permeability, accelerate nitrogen migration and leaching, and lead to problems such as reduced water and nitrogen utilization rates and groundwater pollution (Mokhele et al., 2012; Cameron et al., 2013). Appropriate water and nitrogen supply can produce synergistic and complementary effects, showing the effect of 1 + 1>2 (Fan et al., 2014; Kumar et al., 2016), which can promote crop yield and efficiency while effectively avoiding resource waste and ecological environment problems (Li et al., 2015b; Bai et al., 2018). Cong et al. (2021) conducted a study on the North China Plain and reported that compared with high water and high nitrogen (irrigation of 495 mm and nitrogen application of 330 kg·ha^−1^), the optimal water nitrogen reduction mode (irrigation of 370 mm and nitrogen application of 255 kg·ha^−1^) could improve the soil water storage capacity and soil water content of wheat. NO_3_^−^–N leaching was reduced by 15.87%. Armin et al. (2022) found in a study conducted in Arizona that corn grain yield significantly increased with 90%–100% field capacity (θ_f_) coupled with a nitrogen application of 180 kg·ha^−1^. Rakesh et al. (2022) found in a study conducted in India that the nitrogen uptake of cotton bolls was highest with 600 mm of irrigation and a nitrogen application of 225 kg·ha^−1^, whereas the nitrogen recovery efficiency of cotton reached its peak with 600 mm of irrigation and a nitrogen application of 150 kg·ha^−1^. Cabello et al. (2008) found in a study conducted in Spain that the water use efficiency of melon with 90% crop evapotranspiration combined with 90 kg·ha^−1^ of nitrogen was the highest.

In summary, existing research has focused on water and nitrogen regulation in crops such as wheat, corn, cotton, and other food and cash crops (Zhang et al., 2017; Si et al., 2020; Ishfaq et al., 2018; Emile et al., 2023). Few studies have systematically analyzed the comprehensive effects of water and nitrogen regulation on the production of economic forests and wolfberry, particularly those that consider both production and ecological effects. Gansu Province is the second-largest wolfberry-producing area after the Ningxia Hui Autonomous Region. The Yellow River irrigation region is an important comprehensive agricultural commodity production base in Gansu Province (Yang et al., 2019). Currently, the cultivation area for wolfberry is 36,700 ha, with a production of 20,000 tons of dried fruit. The total output value amounts to two billion yuan, making it an important agricultural industry in the region (Ren and Bai, 2022). However, the characteristics of resource endowment and traditional cultivation practices in the irrigation area of Gansu Province severely hinder the sustainable and healthy development of the wolfberry industry. In view of this, this study used wolfberry as the object aiming to (1) analyze the effects of water and nitrogen regulation on soil water and nitrogen distribution, yield and water-nitrogen use efficiency of wolfberry; (2) clarify the functional relationship between wolfberry productivity and water and nitrogen regulation; (3) correct the wrong cognition of farmers on the water and nitrogen demand law of wolfberry production; (4) obtain the water and nitrogen regulation model for increasing yield and improving efficiency of wolfberry in the Yellow River irrigation region of Gansu Province and other similar climate areas.

## 2 Materials and methods

### 2.1 Description of the experimental site

**Figure 1:**
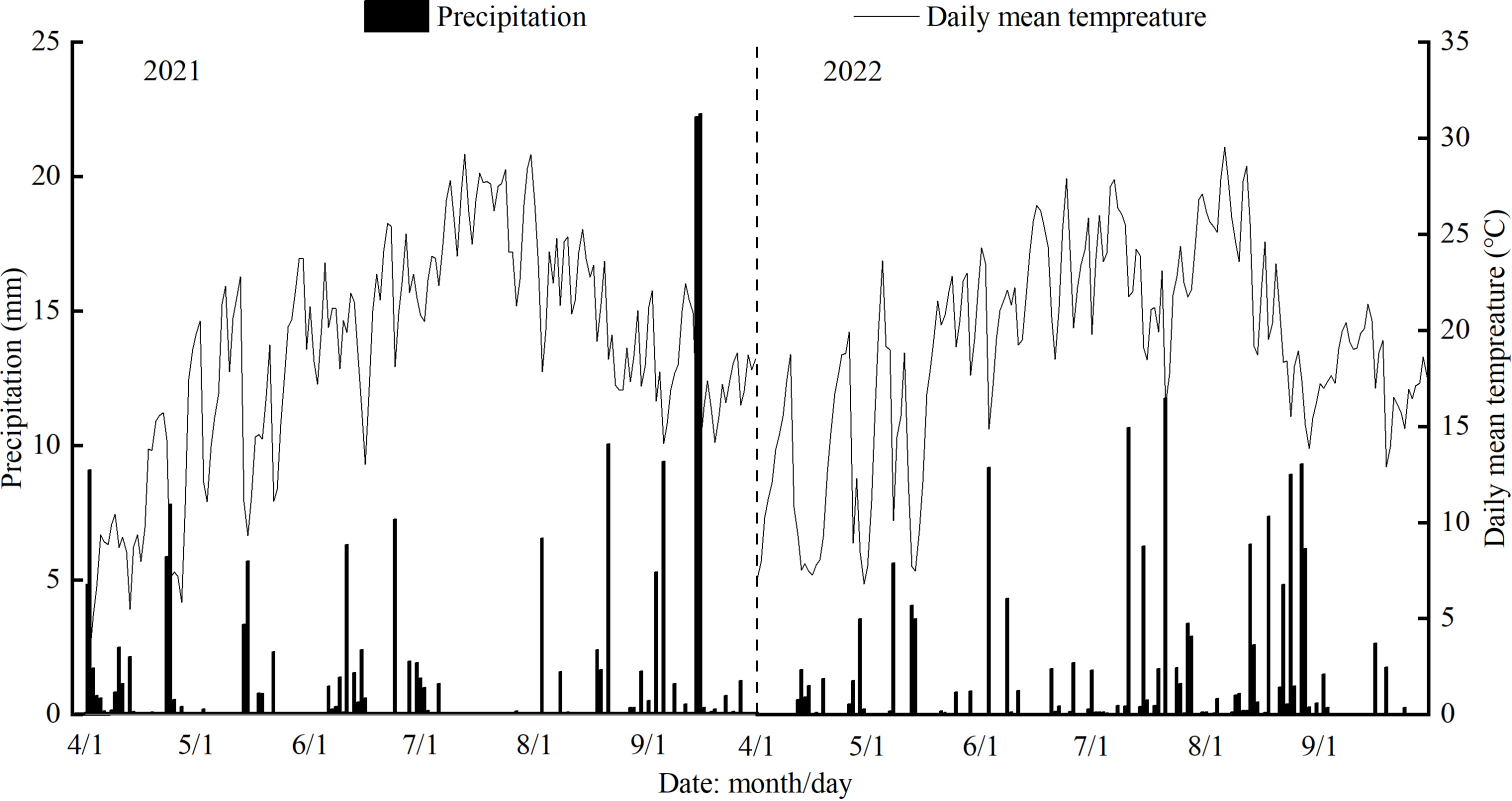
Distribution of precipitation and daily mean temperature during the experiment.

The experiment was conducted at the Irrigation Experimental station (37° 23′ N, 104° 08′ E, altitude 2028 m) of Jingtaichuan Electric Power Irrigation Water Resource Utilization Center in Gansu Province from May to September 2021 and 2022. This region has a temperate continental arid climate, with strong sunshine, rare rainfall, and a dry climate. The annual average sunshine duration, frost-free period, radiation amount, temperature, precipitation, and evaporation are 2652 hours, 191 days, 6.18×10^5^ J·cm^−2^, 8.6 °C, 201.6 mm, and 3028 mm, respectively. The groundwater depth was greater than 40 m. The soil texture of the experimental site was loam, the dry bulk weight of soil was 1.63 g·cm^−3^, and the field water capacity was 24.1% (mass water content). The initial soil properties of the study site was organic matter 6.09 g·kg^−1^, total nitrogen 1.62 g·kg^−1^, total phosphorus 1.32 g·kg^−1^, total potassium 34.03 g·kg^−1^, available nitrogen 74.51 mg·kg^−1^, available phosphorus 26.31 mg·kg^−1^, available potassium 173 mg·kg^−1^, and alkali-hydrolyzed nitrogen 55.2 mg·kg^−1^ in the 0-60 cm soil layer. Meteorological data (precipitation and daily mean temperature) were measured during the experiment using a small intelligent agricultural weather station installed at the test station (**Figure 1**).

### 2.2 Experimental design and field management

The experiment adopted a completely randomized block design, with irrigation and nitrogen application levels as two factors. Among them, the irrigation level (the upper and lower limits of irrigation were set to control the percentage of soil volumetric moisture content to field water capacity θ_f_, and the planned depth of humid layer was 60 cm) included 75%–85% θ_f_ (W0, full irrigation), 65%–75% θ_f_ (W1, slight water deficit), 55%–65% θ_f_ (W2, moderate water deficit) and 45%–55% θ_f_ (W3, severe water deficit); nitrogen application (pure nitrogen) levels included 0 kg·ha^−1^ (N0, no nitrogen application), 150 kg·ha^−1^ (N1, low nitrogen application), 300 kg·ha^−1^ (N2, medium nitrogen application), and 450 kg·ha^−1^ (N3, high nitrogen application) in 16 treatments (**Table 1**). Each treatment was repeated three times for a total of 48 plots. The residential area was 76.5 m^2^ (10.2 m × 7.5 m). The test wolfberry (Ningqi No.5) was a two-year-old seedling transplanted on April 12, 2021, with a plant spacing of 1.5 m and row spacing of 3.0 m. Drip irrigation was then performed. Valves and water meters (accuracy 0.0001 m^3^) were independently installed in the water-delivery pipes of each district to effectively regulate the amount of irrigation (**Table 1**). The spacing of the drip irrigation belt layout was 0.3 m, the design flow rate of the drip head was 2.0 L·h^−1^, and the spacing of the drip head was 0.3 m. In each growing season, nitrogen fertilizer (urea and nitrogen content 46%) according to 6:2:2 was applied during the vegetative growth period (May 16 and 21), the full flowering period (June 4 and 7), and the full fruit period (July 7 and 4). Phosphate (superphosphate, 12% phosphorus content) and potassium (potassium chloride, 60% potassium content) at 130 kg·ha^−1^ were applied as the base fertilizer in a single application during the vegetative growth period (May 16 and 21). The remainder of the field management and pest control were consistent with that of local growers.

**Table 1:** Experimental design.

Treatment	Irrigation level (%)		Nitrogen application level (kg·ha^−1^)	Irrigation quota (mm)	
				2021	2022
W0N0	Full irrigation	75–85	0	316.23	452.84
W0N1			150	360.15	414.34
W0N2			300	324.00	372.16
W0N3			450	302.89	392.60
W1N0	Slight water deficit	65–75	0	286.09	384.91
W1N1			150	312.01	391.40
W1N2			300	279.38	315.41
W1N3			450	278.33	333.27
W2N0	Moderate water deficit	55–65	0	229.70	316.98
W2N1			150	255.67	290.04
W2N2			300	239.30	260.51
W2N3			450	206.89	274.46
W3N0	Severe water deficit	45–55	0	187.28	249.06
W3N1			150	207.54	227.89
W3N2			300	169.31	204.69
W3N3			450	194.62	316.98

### 2.3 Indicators and methods for measurement

#### 2.3.1 Soil moisture content (%)

A portable time-domain reflectometer (TDR; PICO–BT, IMKO, Germany) was used to determine the soil moisture content, irrigation time, and irrigation quota. The TDR tube was placed 0.3 m away from the trunk of the wolfberry in the center of the plot. The observation depth was 120 cm and the measurements were taken every 20 cm. The measurements were performed before and after precipitation and irrigation.

#### 2.3.2 Soil nitrate-nitrogen content (NO_3_^−^–N, mg·kg^−1^)

During the vegetative growth and autumn fruit periods of wolfberry, soil samples were collected using the soil drilling method. Samples were taken from a depth of 0–100 cm at intervals of 10 cm. The collection point was located 0.3 m away from the trunk of the wolfberry in the center of the plot, ensuring a distance from the TDR measuring tube. After air-drying, the soil sample was sieved through a 2 mm screen and extracted with a 2 mol·L^−1^ KCl solution (using a mass ratio of 1:10 for 5 g of dry soil to liquid). The concentration of NO_3_^−^–N in the soil was subsequently measured using a UV-visible spectrophotometer (Beijing Puxi General Instrument Co., Ltd., T6 New Century) (Guo et al., 2022).

(1) Soil-nitrate nitrogen accumulation (*NR*, kg·ha^−1^) (Cambouris et al., 2008).


(1) $$NR=\rho_{i}h_{i}N_{i}/10$$

Where is the bulk density of soil of layer i, g·cm^−3^; *h_i_* is the soil thickness of layer i, cm; *N_i_* is the nitrate nitrogen content of the soil in layer i, mg·kg^−1^.

#### 2.3.3 Wolfberry yield (*Y*, kg·ha^−1^)

Wolfberry was planted in the year in 2021, and there was no yield. This study only analyzed the yield of wolfberry in 2022. From the end of July to the end of August 2022, wolfberry fruits were harvested every 7 days. The weight of the fresh fruit was measured during each harvest, and the sum of the fresh fruit weights represented the total fruit yield for the year. Dried fruits were obtained by natural sun-drying of fresh fruits.

#### 2.3.4 Total nitrogen content of wolfberry fruit (*N_%_*, %)

During the harvest period, three representative wolfberry trees were selected from each plot to pick fresh fruits, which were naturally dried and then baked to a constant quality at 45 °C, crushed, and sifted over 0.5 mm, and then cooked with H_2_SO_4_-H_2_O_2_. The total nitrogen content of the wolfberry fruits was measured using the Kelley nitrogen determination method (Jiang et al., 2022).

#### 2.3.5 Water-nitrogen use efficiency

(1) Water consumption (*ET*, mm) (Nie et al., 2019).

Water consumption during the growth period of the wolfberry was calculated using the water balance method.


(2) ET=P+I+ΔS−U−R−D

Where *P* is the effective rainfall, mm; *I* is the irrigation amount, mm; *ΔS* the change of soil water, mm; *U* is groundwater recharge, mm; *R* is the runoff, mm; *D* is the deep leakage, mm.

Because the groundwater in the test area was buried deep (> 40 m), the terrain was flat, the single rainfall was low, the depth of the wet layer planned by drip irrigation was shallow, and the *U*, *R*, and *D* parameters were ignored.

(1) Water use efficiency (*WUE*, kg·ha^−1^·mm^−1^) (Nie et al., 2019).


(3) WUE=Y/ET

Where *Y* is the yield of wolfberry, kg·ha^−1^; *ET* is the water consumption in 2022, mm.

(2) Nitrogen uptake of fruit (*N_g_*, kg·ha^−1^) (Ding et al., 2023).


(4) $$N_{g}=N_{\%}\times Y$$

Where *N_%_* is total nitrogen content of wolfberry fruit, %.

(3) Nitrogen fertilizer recovery efficiency of fruit (*NRE_g_*, %) (Ding et al., 2023).


(5) $$NRE_{g}=(N_{gl}-N_{g0})/F\times 100\%$$

Where *N_g1_* is the nitrogen uptake of fruit in nitrogen application zone, kg·ha^−1^; *N_g0_* is the nitrogen uptake of fruit in no nitrogen application zone, kg·ha^−1^; *F* is the amount of nitrogen applied, kg·ha^−1^.

### 2.4 Data analysis

Microsoft Excel 2010 was used to organize and calculate the data. IBM SPSS Statistics software (version 25.0) was used for statistical analysis of the data, and one-way ANOVA and Duncan method were used for variance analysis and multiple comparison of indicators in different treatments. Two-factor ANOVA was used to examine irrigation, nitrogen application and their interaction effects, and the significance level α=0.05. Origin 2021 software was used for the drawing.

## 3 Results

### 3.1 Effects of water and nitrogen regulation on temporal and spatial distribution of soil water

**Figure 2:**
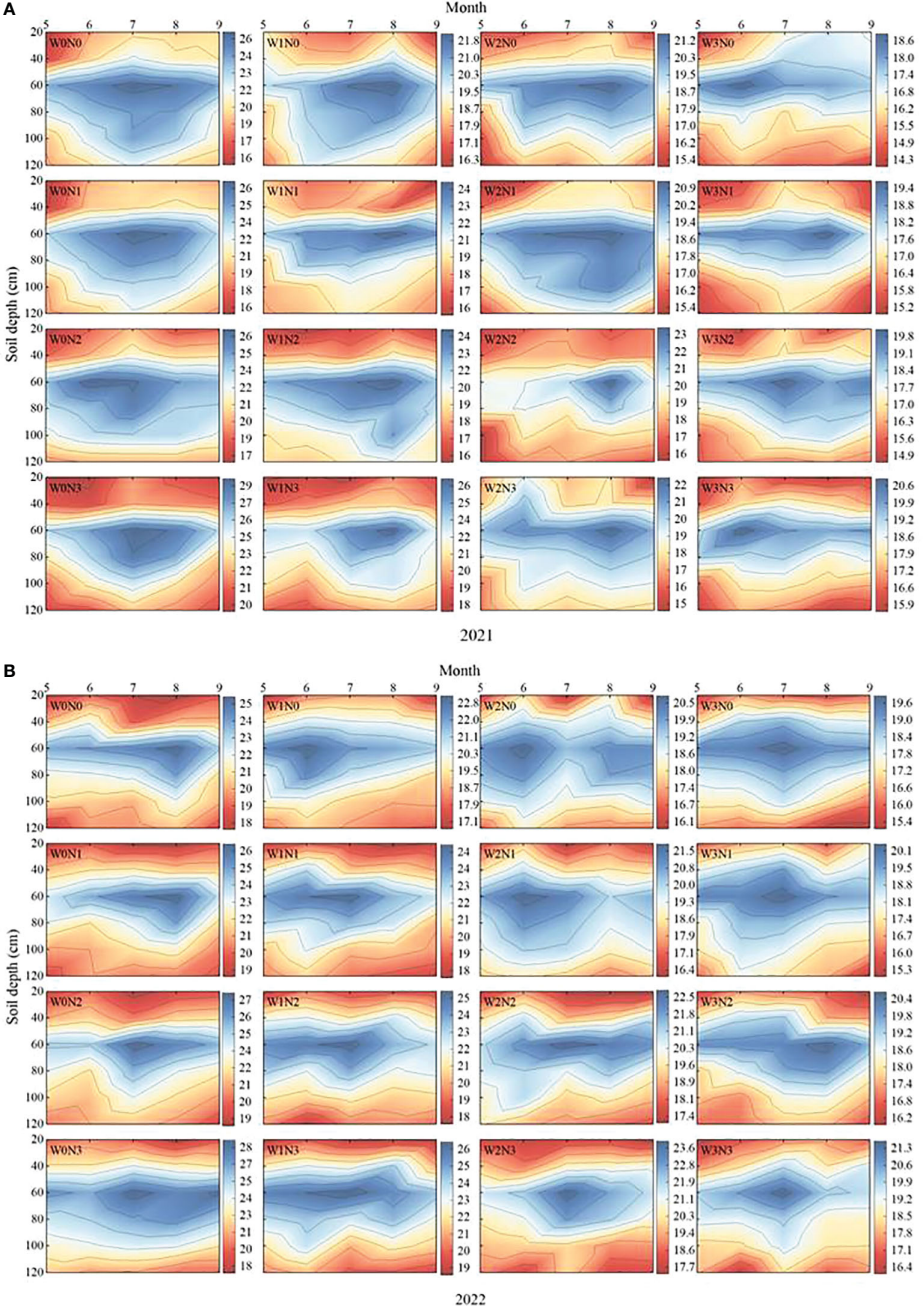
Effects of water-nitrogen regulation on the temporal and spatial distributions of soil water of wolfberry. **(A, B)** represent the temporal and spatial distributions of soil water of wolfberry in 2021 and 2022, respectively. The legends on the right of the figure represents the soil moisture content (%). W0, W1, W2 and W3 refers to full irrigation (75%–85%θ_f_), slight water deficit (65%–75%θ_f_), moderate water deficit (55%–65%θ_f_) and severe water deficit (45%–55%θ_f_), respectively. N0, N1, N2 and N3 refers to the nitrogen application level is 0 kg·ha^−1^, 150 kg·ha^−1^, 300 kg·ha^−1^ and 450 kg·ha^−1^, respectively.

The soil moisture content of the 0–120 cm soil layer first increased and then decreased with the advance of time (growth period) and increase in soil depth (**Figure 2**). In the two-year growing season, the soil moisture content of the 0–40 cm layer changed most obviously with time (growth period), and the overall trend first increased and then decreased. Under the same irrigation level, the soil moisture content followed the order N3 (16.60%–22.93%) > N2 (15.86%–21.49%) > N1 (15.82%–21.39%) > N0 (15.93%–20.45%). Under the same nitrogen application level, the soil moisture content followed the order W0 (18.41%–22.93%) > W1 (16.26%–22.71%) > W2 (16.41%–20.13%) > W3 (15.82%–18.74%). The soil moisture content in the 60–80 cm soil layer decreased significantly with increasing soil depth, and this change was inconsistent with time (growth period). The soil moisture content from June to August (full flowering to full fruit period) was higher than that from May to June (vegetative growth-full flowering period) and from August to September (full fruit-autumn fruit period). The temporal and spatial changes in soil water content were similar in the 40–60 cm and 80–120 cm soil layers, and both first increased and then decreased with the increase in soil depth and advancement of the growth period.

### 3.2 Effects of water and nitrogen regulation on NO_3_^−^–N migration in soil

#### 3.2.1 Distribution of NO_3_^−^–N in soil

**Figure 3:**
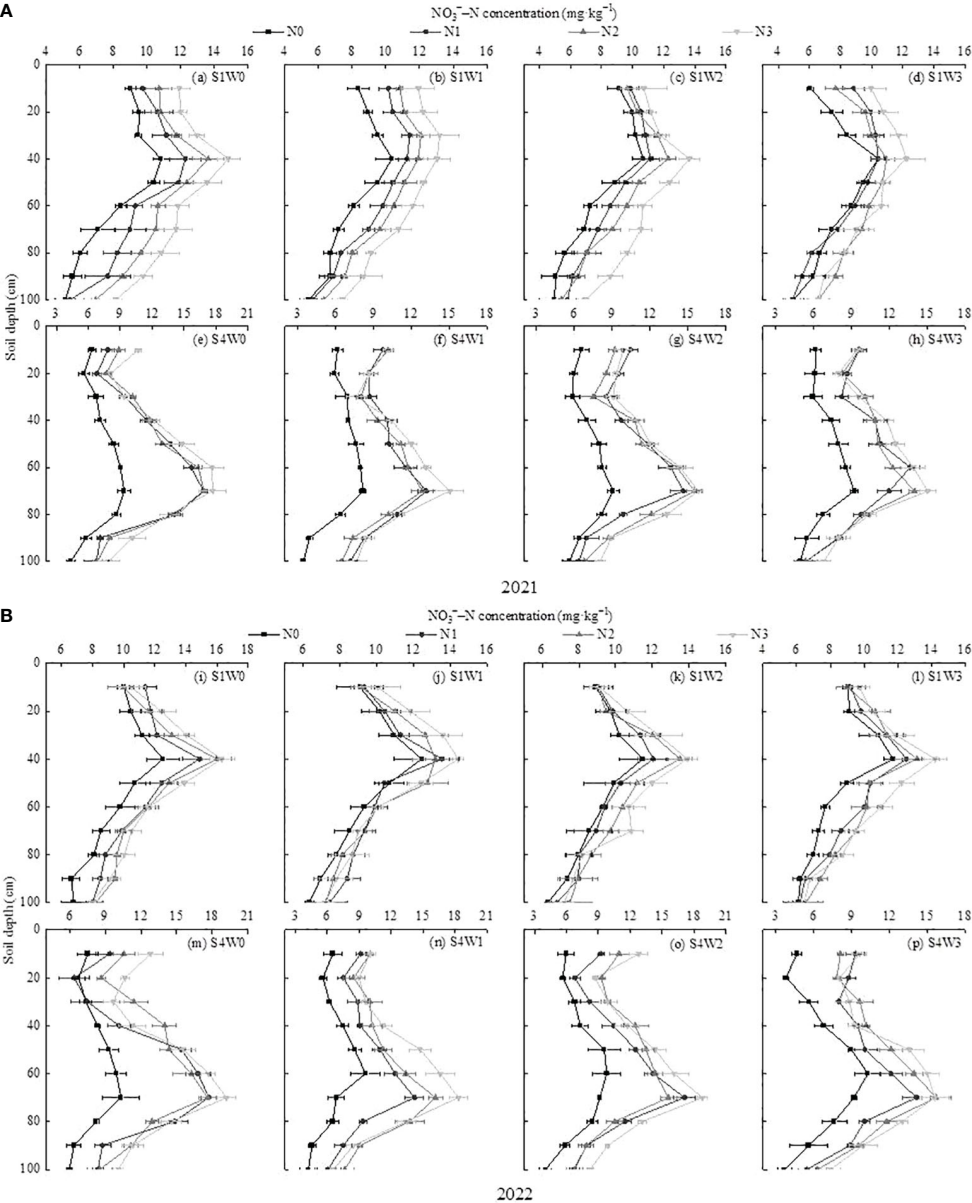
Effects of water and nitrogen regulation on the spatial and temporal distribution of NO_3_^−^–N in the 0–100 cm soil layer. **(A, B)** represent soil NO_3_^−^–N distribution for four irrigation levels in 2021 and 2022, respectively. S1 and S4 correspond to the vegetative growth and autumn fruit periods of wolfberry, respectively.

Vegetative and autumn fruit growth periods are crucial for the overall development and reproductive growth of wolfberry. Taking the vegetative growth and autumn fruit periods as examples, the effects of water and nitrogen regulation on the vertical distribution of soil NO_3_^−^–N were analyzed (**Figure 3**). In the two-year growing season, the NO_3_^−^–N content in the 0–100 cm soil layer in each vegetative growth period first increased and then decreased with increasing soil depth. Under the same irrigation level, the NO_3_^−^–N content in the 0–30 cm soil layer (shallow layer) followed the order N3 > N2 > N1 > N0 with increasing nitrogen application rate. The average NO_3_^−^–N content in N3 was significantly increased by 13.09%–27.66% compared with N0. The NO_3_^−^–N content in the 30–40 cm soil layer showed a leaching peak, and the average NO_3_^−^–N content of N1, N2, and N3 increased by 2.48%–15.92%, 5.48%–26.65%, and 16.64%–33.42%, respectively, compared with N0. The NO_3_^−^–N content in the 40–100 cm soil layer increased with an increase in the nitrogen application rate, and N3 was significantly increased by 22.45%–37.27% compared with N0. Under the same nitrogen application level, the average NO_3_^−^–N content in the 0–100 cm soil layer was W0 > W1 > W2 > W3. W0 increased by 1.92%–21.39%, 4.80%–25.14%, and 10.34%–31.51% compared with W1, W2, and W3, respectively.

In contrast to the vegetative growth period, the NO_3_^−^–N content in the 0–100 cm soil layer in each treatment during the autumn fruit period showed a trend of first decreasing (20–30 cm), increasing (60–70 cm), and then decreasing with increasing soil depth. In the 20–30 cm soil layer, the NO_3_^−^–N content of N0 was significantly lower than that of N1, N2, and N3. The NO_3_^−^–N in the 0–10 cm and 30–100 cm soil layers was in the order N3 > N2 > N1 > N0. The average NO_3_^−^–N content in the 0–100 cm soil layer was N3 > N2 > N1> N0, and N0 decreased by 30.41%–40.42%, 32.97%–42.70%, and 37.36%–47.04% compared with N1, N2, and N3, respectively. Under the same nitrogen application level, the average NO_3_^−^–N content in the 0–100 cm soil layer was W0 > W1 > W2 and W3, and W0 increased by 14.71%–24.40% compared with W1.

#### 3.2.2 Soil NO_3_^−^–N accumulation

Irrigation and nitrogen application had significant effects on NO_3_^−^–N accumulation in the soil during the vegetative growth and autumn fruit periods (*P* < 0.01), and their interaction effects only had significant effects on NO_3_^−^–N accumulation during the autumn fruit period (*P* < 0.01, **Table 2**). During the vegetative growth period, the average accumulation of NO_3_^−^–N in the soil followed the order N3 > N2 > N1 > N0, at the same irrigation level, in which N3 showed an increase of 21.23%–33.47%, 12.21%–16.45%, and 5.86%–9.77% compared with N0, N1, and N2, respectively. At the same nitrogen application level, the average soil NO_3_^−^–N accumulation followed the order W0 > W1 > W2 > W3, and there was no significant difference between W1 and W2 at the N0 and N1 levels.

**Table 2:** Effects of water and nitrogen regulation on soil NO_3_^−^–N accumulation (kg·ha^-1^).

Treatment	2021		Treatment		2022			
	Vegetative growth period	Autumn fruit period			Vegetative growth period		Autumn fruit period	
W0N0	133.16 ± 6.65dA	116.33 ± 5.17cA	W0N0		154.56 ± 8.57bA		130.64 ± 5.85cA	
W0N1	155.44 ± 10.57cA	178.84 ± 9.09bA	W0N1		178.08 ± 7.40aA		186.73 ± 5.73bA	
W0N2	172.24 ± 4.97bA	185.51 ± 6.60bA	W0N2		183.33 ± 5.97aA		203.33 ± 8.32aA	
W0N3	191.80 ± 6.77aA	199.06 ± 11.73aA	W0N3		189.96 ± 3.96aA		215.27 ± 4.29aA	
W1N0	131.75 ± 6.47cA	103.98 ± 4.13cAB	W1N0		150.38 ± 11.41bAB		107.74 ± 7.04dAB	
W1N1	150.53 ± 5.79bA	160.91 ± 7.51bB	W1N1		161.04 ± 3.50abB		154.84 ± 10.37cB	
W1N2	161.45 ± 6.81bB	156.41 ± 3.81abB	W1N2		164.83 ± 9.49abB		179.12 ± 4.43bB	
W1N3	179.54 ± 9.06aAB	170.55 ± 3.55aB	W1N3		171.31 ± 1.80aB		192.21 ± 3.46aB	
W2N0	128.13 ± 10.54cA	115.21 ± 7.06cAB	W2N0		146.26 ± 2.32cAB		117.51 ± 4.87dB	
W2N1	142.22 ± 6.67bcAB	165.48 ± 9.17bB	W2N1		152.70 ± 4.81bcBC		170.38 ± 8.11cBC	
W2N2	149.16 ± 2.75bC	170.40 ± 3.13aC	W2N2		162.01 ± 9.02abB		181.24 ± 2.56bB	
W2N3	172.80 ± 9.14aBC	182.23 ± 4.57aC	W2N3		167.22 ± 7.51aB		201.40 ± 3.92aC	
W3N0	123.47 ± 7.49cA	111.95 ± 6.78bB	W3N0		136.99 ± 4.35cB		108.31 ± 8.87cB	
W3N1	134.46 ± 5.92bcC	159.06 ± 8.73bB	W3N1		150.91 ± 3.89bC		157.12 ± 4.86bC	
W3N2	147.50 ± 2.39abC	162.64 ± 1.84bC	W3N2		157.21 ± 6.22abB		171.61 ± 1.74aB	
W3N3	157.87 ± 11.57aC	172.10 ± 5.99aC	W3N3		164.56 ± 5.12aB		179.84 ± 6.23aD	
Test of variance of significance								
Irrigation (W)	**	**	Irrigation (W)		**		**	
Nitrogen (N)	**	**	Nitrogen (N)		**		**	
W×N	ns	**	W×N		ns		**	

Different lowercase letters indicate the difference between different nitrogen application levels under the same irrigation level, and different capital letters indicate the difference between different irrigation levels under the same nitrogen application level (*P* < 0.05). W and N refer to irrigation and nitrogen application levels, respectively; N × W refers to interaction effect between the two. ** indicates an extremely significant difference (*P* < 0.01); ns indicates no significant difference (*P* > 0.05).

During the autumn fruit period, the average soil NO_3_^−^–N accumulation at the same irrigation level was in the order N3 > N2 > N1 > N0 (except W1). At the W1 level, the cumulative amount of NO_3_^−^–N in the soil during the 2021 growing season was N3 > N1 > N2 > N0, in which N3 increased by 64.02%, 5.99%, and 9.04% compared with N0, N1, and N2, respectively, whereas the cumulative amount of NO_3_^−^–N in the soil during the 2022 growing season followed the order N3 > N2 > N1 > N0. The variation in NO_3_^−^–N accumulation at the same nitrogen application level over the two years was inconsistent, and the growth season in 2021 was in the order W0 > W2 > W3 > W1 (except N1). In the 2022 growing season, the NO_3_^−^–N accumulation was W0 > W2 > W3 > W1 at the N0 and N1 levels, and W0 > W2 > W1 > W3 at the N2 and N3 levels.

### 3.3 Effects of water and nitrogen regulation on the yield of wolfberry

**Figure 4:**
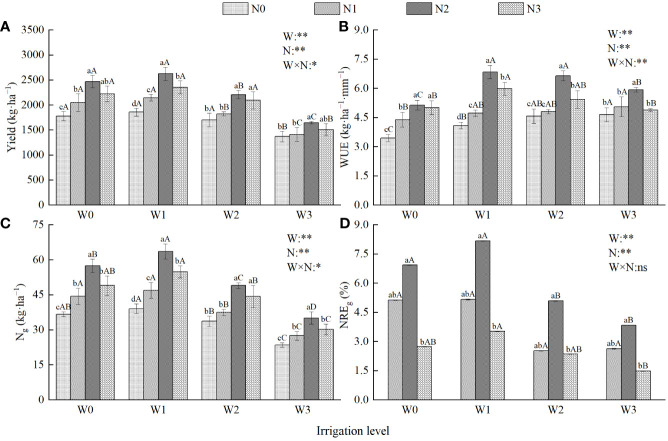
Effects of water and nitrogen regulation on the yield and water-nitrogen use efficiency of wolfberry. **(A–D)** represents yield, water use efficiency, nitrogen uptake and nitrogen recovery efficiency of fruit, respectively. Different lowercase letters indicate the difference between different nitrogen application levels under the same irrigation level, and different capital letters indicate the difference between different irrigation levels under the same nitrogen application level (*P* < 0.05). W and N refer to irrigation and nitrogen application levels, respectively; N × W refers to interaction effect between the two. ** indicates an extremely significant difference (*P* < 0.01); * indicates a significant difference (*P* < 0.05); ns indicates no significant difference (*P* > 0.05).

Irrigation, nitrogen application, and their interaction significantly affected wolfberry yield (*P* < 0.05, **Figure 4A**). Under the same irrigation level, the yield of wolfberry first increased and then decreased with increasing nitrogen application and reached its peak under N2 conditions. The yield of wolfberry significantly increased by 20.38%–41.37%, 16.67%–22.36%, and 5.42%–11.48% compared with N0, N1, and N3, respectively. Under the same nitrogen application level, the yield of wolfberry first increased and then decreased with increasing irrigation amount and reached its peak under W1 conditions; the yield was significantly increased by 4.41%–6.36%, 9.23%–18.97%, and 35.67%–59.26% compared with W0, W2, and W3, respectively. Among all treatments, W1N2 had the highest yield of wolfberry (2623.09 kg·ha^−1^).

**Figure 5:**
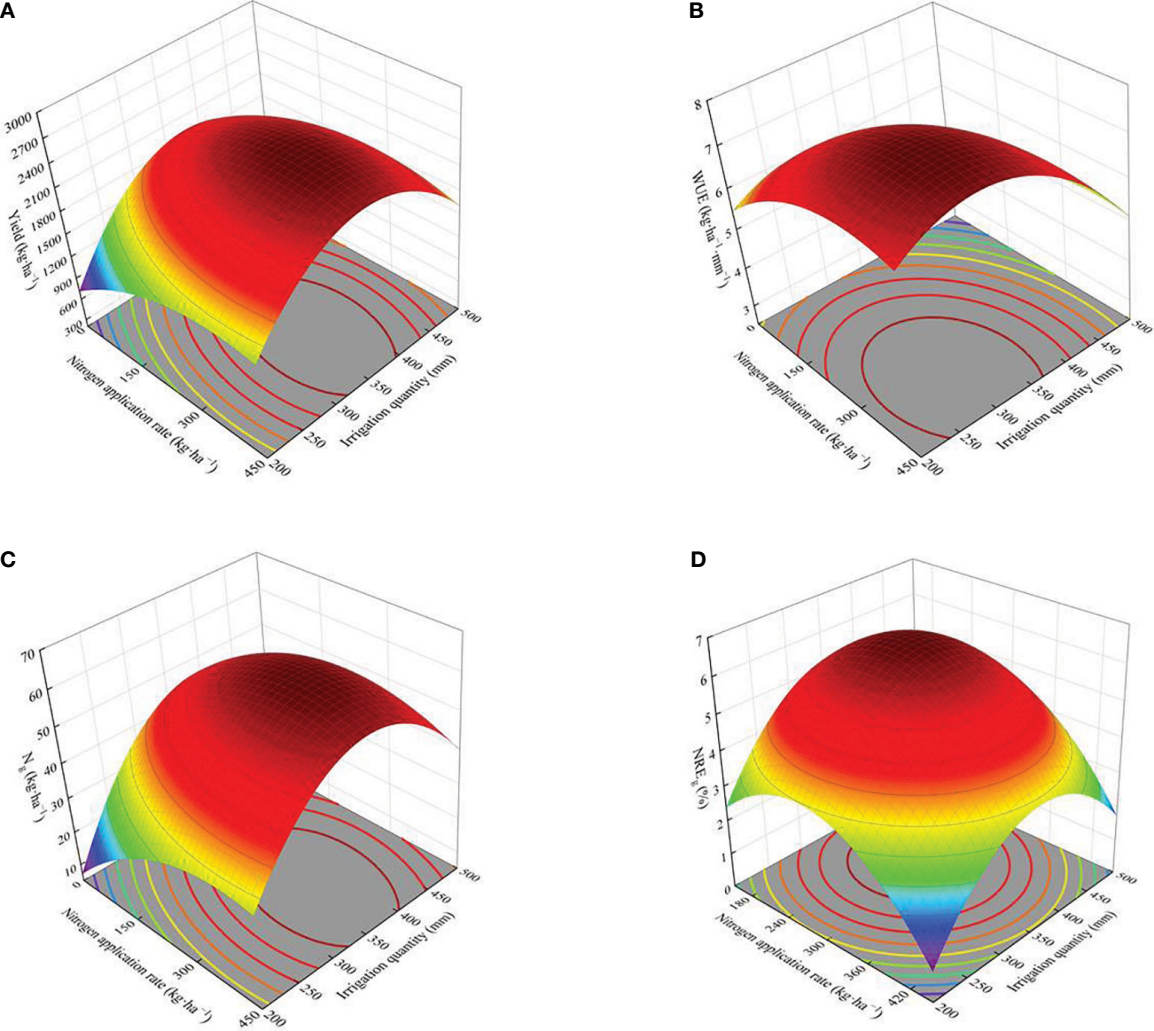
Regression models of wolfberry yield and water-nitrogen use efficiency under different water and nitrogen regulations. **(A–D)** represents yield, water use efficiency, nitrogen uptake and nitrogen recovery efficiency of fruit, respectively.

Regression analysis was conducted with the irrigation amount (*W*) and nitrogen application amount (*N*) as independent variables and yield (*Y*) as the dependent variable (**Table 3** and **Figure 5A**), and the fitting equation was obtained: *Y*=-0.037*W^2^*-0.006*N^2^*-0.004*WN*+28.164*W*+5.155*N*-3439.08, *P* < 0.01, and *R^2 = ^* 0.878. This shows that the regression equation has a good fit and a high prediction reliability. The coefficient of the primary term of the equation was positive, and the coefficient of the secondary term was negative, indicating that the yield of wolfberry first increased and then decreased with an increase in the irrigation and nitrogen application, whereas the coefficient of the interaction term of water and nitrogen was negative, and the interaction between the two was significant (**Figure 4A**), indicating that there was a significant coupling effect between water and nitrogen yield. When the yield of wolfberry was the highest, and the corresponding amounts of irrigation and nitrogen application amount were 363.93 mm and 308.27 kg·ha^−1^, respectively.

**Table 3:** Regression equations of yield and water-nitrogen use efficiency of wolfberry under different water and nitrogen treatments.

Dependent variable	Regression equation	*R^2^*	*P*
Yield (*Y*)	*Y*= –0.037*W^2^*–0.006*N^2^*–0.004*WN*+28.164*W*+5.155*N*–3439.08	0.878	< 0.01
Water use efficiency (*WUE*)	*WUE*= –0.000061*W^2^*–0.000014*N^2^ + *0.000002*WN*+0.036*W*+0.009*N*+0.678	0.731	< 0.01
Nitrogen uptake of fruit (*N_g_*)	*N_g_* = –0.001*W^2^*–0.00018*N^2^*–0.00015*WN*+0.794*W*+0.165*N*–111.696	0.854	< 0.01
Nitrogen fertilizer recovery efficiency of fruit (*NRE_g_*)	*NRE_g_* = –0.000097*W^2^*–0.000085*N^2^*–0.000005*WN*+0.073*W*+0.047*N*–13.333	0.745	< 0.01

### 3.4 Effects of water and nitrogen regulation on water-nitrogen use efficiency of wolfberry

#### 3.4.1 Water consumption characteristic

**Figure 6:**
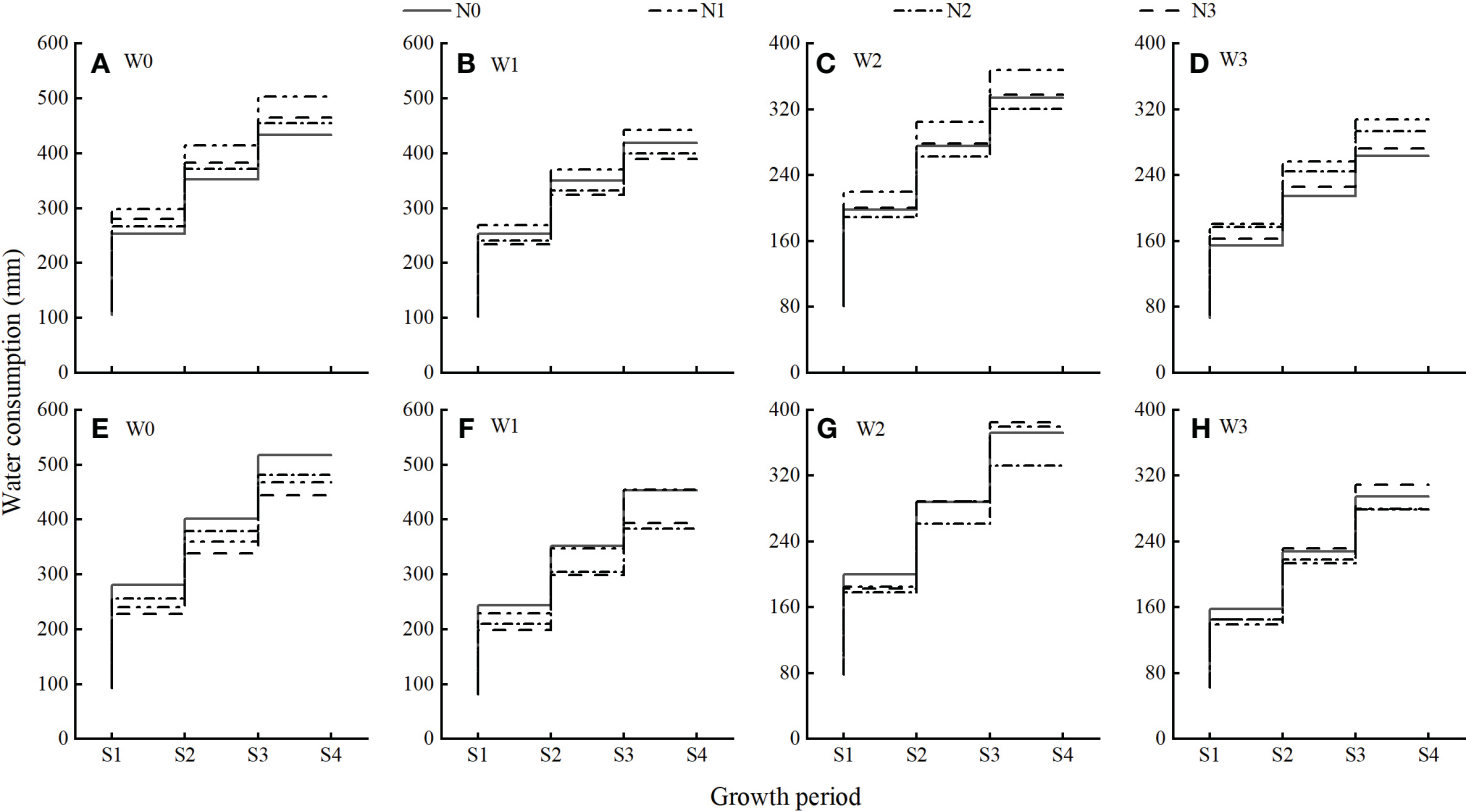
Water consumption during different growth periods of wolfberry under different water and nitrogen treatments. (S1) – (S4) correspond to the vegetative growth, full flowering, full fruit, and autumn fruit periods, respectively. **(A–D)** is the water consumption for each growth period in 2021, and **(E–H)** is the water consumption for each growth period in 2022.

As shown in **Figure 6**, under different water and nitrogen treatments in the 2021 growing season, the water consumption and the proportion of water consumption in the vegetative growth period, full flowering period, full fruit period, and autumn fruit period were 66.68–129.74 mm and 24.49%–28.16%, 88.12–170.42 mm and 22.05%–24.66%, 60.16–115.98 mm and 30.60%–33.85%, and 46.90–88.91 mm and 16.57%–18.62%, respectively; the total water consumption was the highest in W0N1 treatment (503.42 mm) and the lowest in W3N0 treatment (263.27 mm). Compared with the growing season in 2021, the water consumption during the vegetative growth, full flowering, and full fruit periods of the growing season in 2022 under different water and nitrogen treatments decreased by 1.28%, 2.16%, and 6.36%, respectively, whereas the water consumption in the autumn fruit period increased by 30.85%. The total water consumption was highest (517.17 mm) in the W0N0 treatment and lowest (278.57 mm) in the W3N2 treatment.

#### 3.4.2 Water use efficiency

The water use efficiency of wolfberry was significantly affected by irrigation, nitrogen application, and their interaction (*P* < 0.01, **Figure 4B**). Under the same irrigation level, the water use efficiency of wolfberry was in the order N2 > N1 > N3 > N0, in which N2 was significantly increased by 27.31%–66.99%, 16.86%–44.70% and 2.60%–22.28% compared with N0, N1, and N3, respectively. Under the same nitrogen application level, the water use efficiency of wolfberry showed different trends with increasing irrigation amount. At the N0 and N1 levels, the water use efficiency of wolfberry decreased with an increase in irrigation amount, and the difference between W0 and W3 was significant. Under N2 and N3 levels, the water use efficiency of wolfberry first increased and then decreased with increasing irrigation amount. Among all treatments, W1N2 had the highest water use efficiency (6.83 kg·ha^−1^·mm^−1^).

The determination coefficient of the fitting equation for the water use efficiency of wolfberry was 0.731 (*P* < 0.01, **Table 3** and **Figure 5B**), indicating that irrigation and nitrogen application had significant effects on the water use efficiency of wolfberry (**Figure 4B**). In the model, the coefficients of the primary and secondary terms of irrigation water quantity were positive and negative, respectively, indicating that water use efficiency first increased and then decreased with the increase in irrigation water quantity, which was inconsistent with the change rule of water use efficiency decreasing with the increase in irrigation water quantity under N0 and N1 levels (**Figure 4B**); therefore, the optimization of the model failed.

#### 3.4.3 Nitrogen use efficiency

##### 3.4.3.1 Nitrogen uptake of fruit

Irrigation, nitrogen application, and their interaction significantly affected the nitrogen uptake of wolfberry fruits (*P* < 0.05, **Figure 4C**). Under the same irrigation level, the nitrogen absorption of wolfberry fruit was N2 > N1 > N3 > N0, and N2 was significantly increased by 45.37%–62.77%, 27.59%–35.70%, and 10.62%–17.40% compared with N0, N1, and N3, respectively. Under the same nitrogen application level, the nitrogen absorption of wolfberry fruit was W1 > W0 > W2 > W3, and W1 significantly increased by 5.64%–12.16%, 15.94%–29.82%, and 65.96%–81.81% compared with W0, W2, and W3, respectively. In all treatments, the nitrogen uptake of W1N2 fruits reached a peak of 63.56 kg·ha^−1^.

##### 3.4.3.2 Nitrogen fertilizer recovery efficiency of fruit

Irrigation and nitrogen application had significant effects on the nitrogen recovery efficiency of fruits (*P* < 0.01), but their interaction had no significant effect on the nitrogen recovery efficiency of fruits (*P* > 0.05, **Figure 4D**). Under the same irrigation level, the nitrogen recovery efficiency of fruit was higher than that of N1 and N3, and N2 was significantly increased by 35.09%–101.98% and 116.60%–159.46% compared with N1 and N3, respectively. Under the same nitrogen application level, the nitrogen recovery efficiency of fruit was in the order W1 > W0 > W2 > W3, and W1 significantly increased by 0.58%–28.94%, 49.79%–104.76%, and 96.20%–137.84% compared with W0, W2, and W3, respectively. In all treatments, the nitrogen recovery efficiency of W1N2 fruit peaked at 8.17%.

The coefficient of the primary term of nitrogen uptake (**Table 3** and **Figure 5C**) and fruit nitrogen recovery efficiency model (**Table 3** and **Figure 5D**) were positive, whereas that of the secondary term was negative, indicating that both first increased and then decreased with increasing irrigation and nitrogen application. The interaction coefficient was negative, indicating a significant interaction between fruit nitrogen uptake (**Figure 4C**). These results indicate that there is a certain coupling between water-nitrogen and nitrogen uptake and the nitrogen recovery efficiency of the fruit. However, this is inconsistent with the result that the interaction between the nitrogen recovery efficiency of fruit is not significant (**Figure 4D**), and the model of nitrogen recovery efficiency of fruit cannot be optimized; therefore, only the regression model fort fruit nitrogen uptake is optimized. The results showed that when the irrigation amount was 374.31 mm and the nitrogen application amount was 302.37 kg·ha^−1^, the results of the optimization were as follows: the wolfberry fruit had the highest nitrogen absorption (61.84 kg·ha^−1^).

## 4 Discussion

### 4.1 Effects of water and nitrogen regulation on temporal and spatial distribution of soil moisture in wolfberry

Soil moisture is a key factor constraining agroforestry production, which is closely related to plant physiological growth and yield accumulation, and its influence is particularly pronounced in arid and semi-arid regions where water resources are scarce (Liu et al., 2020a). On the one hand, irrigation and nitrogen application are two interconnected factors that influence the soil moisture status. Moisture can mobilize the enthusiasm of cell turgor, promote the division and extension of plant cells and affect the growth and redistribution of plant roots (Tang et al., 2022). After the irrigation satisfies the normal water demand and evapotranspiration of the plants, excessive moisture will directly affect the spatial and temporal distribution of soil moisture by altering the soil structure, water-holding and water-conducting capacity (Hu et al., 2011). On the other hand, by participating in plant cell metabolism to enhance the water absorption capacity of plant roots, and increase soil water potential and activating deep soil water, nitrogen can indirectly affect the spatial and temporal distribution of soil water (Hu et al., 2011; Wu et al., 2018). At the same time, appropriate water and nitrogen regulation can effectively increase soil available nitrogen content, promote plant organic synthesis, and make up for the negative effects of external factors on crop growth. In this study, it was found that the soil water content of wolfberry was maintained at 14.08%–29.03% throughout the reproductive period under different water and nitrogen regulations. The 0–40 cm soil layer exhibited the most active soil water content, with an overall performance of W0 > W1 > W2 > W3 and N3 > N2 > N1 > N0. This finding was similar to those reported by Zheng et al. (2012) and Zhan et al. (2016). The active layer of the wolfberry root system is primarily concentrated at a depth of 0–40 cm (Zhan et al., 2016). The soil water content is significantly affected by irrigation and crop root activities, and the root system of wolfberry within this soil layer is well-developed and exhibits a strong water absorption capacity (Hupet and Vanclooster, 2002). However, Yin et al. (2021) found that the soil water content of the 0-60 cm soil layer of wolfberry varied significantly under different irrigation rates in the gray-calcium soil area of Ningxia. This may be related to the soil texture in the test area, which is characterized by a low-water holding capacity in the field and a high infiltration coefficient in the profile (Jin and Ma, 2000), which is prone to deep soil seepage. Consistent with the findings of Zhao et al. (2021) regarding various sand-resistant vegetation in the Mu Us Sandy area, this study also observed an “S-shaped” change in the soil water content in the 0-100 cm soil layer. This is mainly because crop growth relies primarily on the consumption of shallow soil moisture. Compared with shallow soil, deep soil is less disturbed by tillage and is more stable (Kang et al., 2023), and the water not absorbed by the crop root system will naturally infiltrate under the influence of gravity (Jia et al., 2013), causing water to accumulate in a specific soil layer.

### 4.2 Effects of water and nitrogen regulation on soil NO_3_^−^–N distribution in wolfberry

The soil NO_3_^−^–N content, the main inorganic form of nitrogen that is easily absorbed and utilized by crops, represents the ability of soil to supply nitrogen to a certain extent, which is of decisive significance for the improvement of soil fertility, promotion of crop growth, and enhancement of economic output (Darren et al., 2020). In this study, the average NO_3_^−^–N content in the 0–100 cm soil layer during the nutrient growth period of wolfberry was in the order of W0 > W1 > W2 > W3 and N3 > N2 > N1 > N0, suggesting that soil NO_3_^−^–N transport and crop nitrogen uptake are susceptible to the effects of irrigation and nitrogen application (Emile et al., 2023). Meanwhile, excessive irrigation and nitrogen application cannot be sufficiently absorbed and utilized by crop roots, which may cause soil nitrogen leaching loss, reduce nitrogen use efficiency, and increase the risk of soil compaction and secondary salinization (Luo et al., 2020). Guo et al. (2022) concluded that soil NO_3_^−^–N content in the 0–200 cm soil layer of vineyards showed a gradual increase from top to bottom in sandy loam soil in the Taihang Mountain region of Hebei Province. However, in this study, conducted on loam soil in the Yellow Irrigation Area of Gansu Province, it was observed that the NO_3_^−^–N content in the 0–100 cm soil layer of wolfberry showed an increase followed by a decrease during the nutrient growth period and a decrease followed by an increase followed by a subsequent decrease during the fall fruiting period. Sandy loam has larger particles, extreme water permeability, very poor water and fertilizer retention capacity, and is prone to oxidize NH_4_^+^ to NO_3_^−^, increasing the risk of nitrogen leaching. Gao et al. (2005) observed no significant correlation between spring corn soil NO_3_^−^–N accumulation and irrigation volume in Yongshou County, Shaanxi Province. However, we found that the average soil NO_3_^−^–N accumulation during the nutritive growth and fall fruiting periods of wolfberry was W0 > W1 > W2 > W3 and W0 > W2 > W1 and W3, respectively. The results showed that compared with adequate irrigation, proper control of irrigation amount was conducive to reducing the accumulation of soil inorganic nitrogen, reducing the risk of NO_3_^−^–N leaching (Lv et al., 2018), increasing the rate of soil mineralization, promoting soil ecological balance and organic matter decomposition, and improving soil fertility and crop nutrient supply (Zheng et al., 2020). At the same time, the soil NO_3_^−^–N accumulation of W0N1, W0N2 and W0N3 treatments showed a trend of increasing gradually with the increase of nitrogen application. The result indicates that under the condition of high nitrogen application rate, excessive irrigation can easily cause NO_3_^−^–N leaching in soil, increase the concentration of soil solution, enhance the nitrification and denitrification of soil microorganisms, and lead to soil acidification and greenhouse gas emissions (Chen et al., 2019). In addition, this study found that under different water and nitrogen regulations (except N0), soil NO_3_^−^–N accumulation was higher during the autumn fruit period of wolfberry than during the vegetative growth period. Compared with the pre-reproductive stage, wolfberry had a lower demand for water and nitrogen during the late reproductive growth period. Excessive nitrogen content in the soil increases the substrate concentration of nitrification and promotes nitrification fluxes, and excessive soil moisture exacerbates the migration and leaching of residual nitrogen (Liu et al., 2020b), which increases the risk of groundwater contamination.

### 4.3 Effects of water and nitrogen regulation on the yield of wolfberry

Crop yield is one of the most intuitive indexes to evaluate planting efficiency, and it is closely related to soil water and fertilizer status. Excessive irrigation and fertilization application may cause the phenomena of “futile growth” and “low flowering and low yield”, which may produce deep soil leakage and reduce the utilization rate of water and fertilizer resources (Haefele et al., 2008; Min et al., 2011). Reasonable water and fertilizer supply can achieve the effect of “regulating fertilizer with water and promoting water with fertilizer” to realize a synergistic effect on crop yield (Er et al., 2022). In this study, the yield of wolfberry was W1 > W0 > W2 > W3 and N2 > N3 > N1 > N0. This is consistent with the findings of Liu et al. (2018a) in the Inner Mongolia Autonomous Region and Ma et al. (2023) in the central arid region of Ningxia on wolfberry. The result showed that when irrigation and nitrogen supply are coordinated, it can maintain appropriate soil moisture and nutrient concentrations, increase the reproduction of soil ammonia bacteria and soil ammonium nitrogen content, and ultimately promote the accumulation of crop dry matter and yield formation (Ihsan et al., 2022). The study revealed that the water and nitrogen inputs required to achieve the highest wolfberry yield varied by region. Specifically, the Yellow Irrigation Area of Gansu Province demonstrated the highest yield (2623.09 kg·ha^−1^) when the irrigation-applied nitrogen levels were 315.41 mm and 300 kg·ha^−1^, respectively. The highest yield (5547.22 kg·ha^−1^) was obtained in the Inner Mongolia Autonomous Region when the nitrogen applied by irrigation was 750 kg·ha^−1^ and 285.00 mm, respectively. Similarly, the highest yield (2356.34 kg·ha^−1^) was obtained in the central arid area of Ningxia when the nitrogen applied by irrigation was 225 kg·ha^−1^ and 256.50 mm, respectively. In addition, we found that the wolfberry yield first increased and then decreased when the lower limit of irrigation was reached. This is inconsistent with the results of Wang et al. (2015), who concluded that the yield of wolfberry significantly increased with an increase in the irrigation lower limit in their study conducted in Qinghai. This may stem from the following two factors: one is related to the age of the wolfberry (2–3 years and 3–4 years, respectively); as the wolfberry plants grow, their growth increases, leading to an increased demand for water. The other factor is related to the climate of the test site, which can be either a temperate continental arid climate or a plateau continental climate. Compared with the temperate continental arid climate, the plateau continental climate has stronger solar radiation and higher soil evaporation. As a result, wolfberry growth is more dependent on irrigation.

### 4.4 Effects of water and nitrogen regulation on water-nitrogen use efficiency of wolfberry

Water and nitrogen utilization efficiency not only reflects the energy conversion in the crop production process but also measures the suitability of crop growth and yield-to-input ratio (Qin et al., 2021). Studies have shown that the water use efficiency of corn and wheat tends to increase and then decrease with an increase in irrigation and nitrogen application, whereas the nitrogen fertilizer recovery efficiency tends to increase and then decrease with an increase in irrigation (Liu et al., 2018b; Wang et al., 2022). However, in this study, we found that the water use efficiency of wolfberry decreased with increasing irrigation at the N0 and N1 levels, and the nitrogen fertilizer recovery efficiency of fruits decreased, then increased, and then decreased with increasing irrigation at the N1 level. Excessive irrigation leads to soil nutrient leaching, reduces soil fertility, and simultaneously changes crop cell expansion pressure, weakening the ability of crops to absorb nitrogen and resulting in crop yield reduction, thus affecting water use efficiency and fruit nitrogen fertilizer recovery efficiency. This study showed that the nitrogen uptake of wolfberry fruits first increased and then decreased with increasing nitrogen application. This is inconsistent with the findings of Luo et al. (2020) in the Shaanxi area, who showed that the nitrogen uptake of 100 kg of winter wheat grain increased with an increase in nitrogen application. This may be related to the nitrogen application gradient settings. Wheat is a gramineous grain that is significantly affected by exogenous nitrogen (Lv et al., 2023). Although herbaceous wolfberry plants obtain inherent nitrogen from the soil, their roots can absorb a large amount of exogenous nitrogen, which promotes the transfer of nitrogen stored in the vegetative organs to the fruits and increases the total nitrogen content of the fruits, thereby increasing their nitrogen uptake. In addition, in this study, the change in water use efficiency of wolfberry and the amount of irrigation water (**Figure 4B**) was inconsistent with the constructed model (**Table 3**). Similarly, the change in fruit nitrogen fertilizer recycling efficiency with the amount of irrigation water and the response to water and nitrogen (**Figure 4D**) was inconsistent with the constructed model (**Table 3**). This phenomenon may be because the irrigation and nitrogen application levels set in this experiment did not reach the threshold for water-use efficiency. Compared with N1, the increase in fruit nitrogen uptake in N2 was lower than that in nitrogen application, which led to an inconsistency between the change rule of fruit nitrogen fertilizer recycling efficiency with the amount of irrigation in the model and the experimental results. As a result, the model failed to achieve optimal success. Therefore, in production practice, comprehensive consideration should be given to crop dry matter accumulation as well as water and nitrogen utilization efficiency. Therefore, it is important to adopt reasonable water and nitrogen management modes.

## 5 Conclusions

During the two-year wolfberry growing season, the average soil moisture content followed the order W0 > W1 > W2 > W3 and N3 > N2 > N1 > N0, depending on the nitrogen application and irrigation amount. The average water consumption during the full flowering period accounted for 24.36%–27.46% of the total water consumption. During the vegetative growth period, the soil NO_3_^−^–N content of different water-nitrogen combinations increased and then decreased with increasing soil depth, with average NO_3_^−^–N accumulation ranging from 130.23–190.88 kg·ha^-1^. During the autumn fruiting period, there was a decrease, followed by an increase and then a decrease, with the average accumulation ranging from 123.49–207.17 kg·ha^−1^. The wolfberry yield followed the order W1 > W0 > W2 > W3 and N2 > N3 > N1 > N0. The wolfberry yield was obtained when treated with W1N2, which was 18.04%, 18.97%, and 85.80% higher than those treated with W0N3, W2N2, and W3N1, respectively. The water use efficiency, fruit nitrogen uptake, and fruit nitrogen recovery efficiency first increased and then decreased with increasing nitrogen application. The results of the regression analysis revealed that the optimal combined effects of wolfberry yield, water use efficiency, and nitrogen use efficiency were within the range of 315.4–374.3 mm, and the nitrogen application rate ranged from 300.0–308.3 kg·ha^−1^ in the Yellow River Irrigation Region of Gansu Province. The present findings contribute novel insights and theoretical bases for water and nitrogen management of wolfberry in the Yellow River Irrigation Region. However, climatic factors (such as rainfall) and soil conditions (such as field water capacity, soil texture) vary in different region. Therefore, the mechanisms of climate change and soil conditions in different regions influencing the effects of water and nitrogen regulation on soil water and nitrogen distribution, yield and water and nitrogen use efficiency of wolfberry require further investigation.

## Data availability statement

The original contributions presented in the study are included in the article/supplementary material. Further inquiries can be directed to the corresponding author.

## Author contributions

RT: Conceptualization, Data curation, Formal Analysis, Investigation, Methodology, Writing – original draft. GQ: Conceptualization, Formal Analysis, Funding acquisition, Writing – review & editing. YK: Data curation, Formal Analysis, Project administration, Writing – review & editing. QJ: Funding acquisition, Writing – review & editing. JW: Supervision, Writing – review & editing. FX: Writing – review & editing. YG: Investigation, Writing – review & editing. CW: Methodology, Writing – review & editing. QL: Project administration, Writing – review & editing. QC: Project administration, Writing – review & editing.
